# Prenatal Diagnosis and Postnatal Management of Congenital Cervicofacial Cystic Lymphangioma Associated With Multiple Fetal Anomalies: A Case Report

**DOI:** 10.7759/cureus.110357

**Published:** 2026-06-06

**Authors:** Anibri Mouna, Khaoula Lakhdar, Ibtissam Bensghir, Fethi Khalid

**Affiliations:** 1 Department of Obstetrics and Gynecology, Oncology and High-Risk Pregnancy, Souissi Maternity Hospital, Rabat, MAR; 2 Department of Gynecology-Obstetrics and Endoscopy, Souissi Maternity Hospital, Rabat, MAR; 3 Department of Gynecology-Obstetrics and Endoscopy, Ibn Sina University Hospital Center, Mohammed V University, Rabat, MAR; 4 Department of Gynecology, Souissi Maternity Hospital, Rabat, MAR

**Keywords:** congenital lymphatic malformation, cystic hygroma, cystic lymphangioma, fetal ultrasound, prenatal diagnosis

## Abstract

Cystic lymphangioma is a rare benign congenital malformation of the lymphatic system resulting from abnormal embryologic development of lymphatic vessels. The cervicofacial region represents the most common localization. Prenatal ultrasound plays a key role in early diagnosis and follow-up. We report the case of a 30-year-old gravida 2 para 1 woman with a pregnancy complicated by primary cytomegalovirus (CMV) infection during the first trimester and multiple fetal anomalies identified on antenatal ultrasound, including ventriculomegaly, cerebellar hypoplasia, abnormal cerebral gyration, cardiomegaly, and cystic hygroma. The pregnancy was followed up with serial ultrasounds until term. Vaginal delivery occurred at 39 weeks of gestation without maternal complications. The neonate presented with a cervicofacial cystic mass and was referred for pediatric surgical management. Histopathological examination after surgical excision confirmed the diagnosis of cystic lymphangioma. This case highlights the importance of prenatal imaging in the diagnosis and multidisciplinary management of congenital lymphatic malformations.

## Introduction

Cystic lymphangioma, also known as cystic hygroma, is a rare benign congenital lymphatic malformation arising from abnormal development of the primitive lymphatic system [1]. It is characterized by cystic dilatation of lymphatic vessels caused by a failure of communication between lymphatic sacs and the venous system during embryogenesis. Approximately 75% to 80% of cases occur in the cervicofacial region, especially in the posterior cervical triangle and submandibular spaces [2].

Prenatal diagnosis has become increasingly frequent due to the widespread use of obstetrical ultrasonography [3]. Ultrasound examination typically reveals a multiloculated cystic mass with thin septations [4]. Some cases are associated with chromosomal abnormalities, congenital infections, hydrops fetalis, or other structural fetal anomalies.

The evolution of cystic lymphangioma is variable, ranging from spontaneous regression to progressive enlargement leading to airway compromise or cosmetic deformity. Management generally requires multidisciplinary care involving obstetricians, neonatologists, radiologists, pediatric surgeons, and pathologists.

We present a case of prenatally diagnosed cervicofacial cystic lymphangioma associated with multiple fetal anomalies in a term pregnancy.

## Case presentation

A 30-year-old woman, gravida 2 para 1, with no significant past medical or surgical history, was followed at the maternity unit of Ibn Sina University Hospital in Rabat, Morocco. She had no history of oral contraceptive use or consanguinity. Her blood group was A positive.

The current pregnancy was estimated at 39 weeks of gestation at admission. Prenatal investigations revealed a positive cytomegalovirus (CMV) serology during the first trimester, likely contracted from her two-year-old child. Due to financial limitations, amniocentesis was not performed between 18 and 20 weeks of gestation.

A monthly ultrasound follow-up was carried out throughout pregnancy. The second-trimester morphological ultrasound identified several fetal abnormalities, including ventriculomegaly, a cerebellum smaller than expected for gestational age, abnormal cerebral gyration, cerebral parenchymal atrophy, cardiomegaly with situs solitus, and a cystic hygroma located in the cervicofacial region.

No specific treatment was administered during pregnancy.

The patient was admitted at 39 weeks of gestation in spontaneous labor. On admission, she was in good general condition, afebrile, normotensive, with a body mass index of 27 kg/m². Urine dipstick testing was negative.

Obstetrical examination revealed a uterine height of 30 cm and regular fetal heart sounds. Vaginal examination demonstrated an 80% effaced cervix dilated to approximately 2 cm, intact membranes, cephalic presentation, and a clinically adequate pelvis.

**Figure 1:**
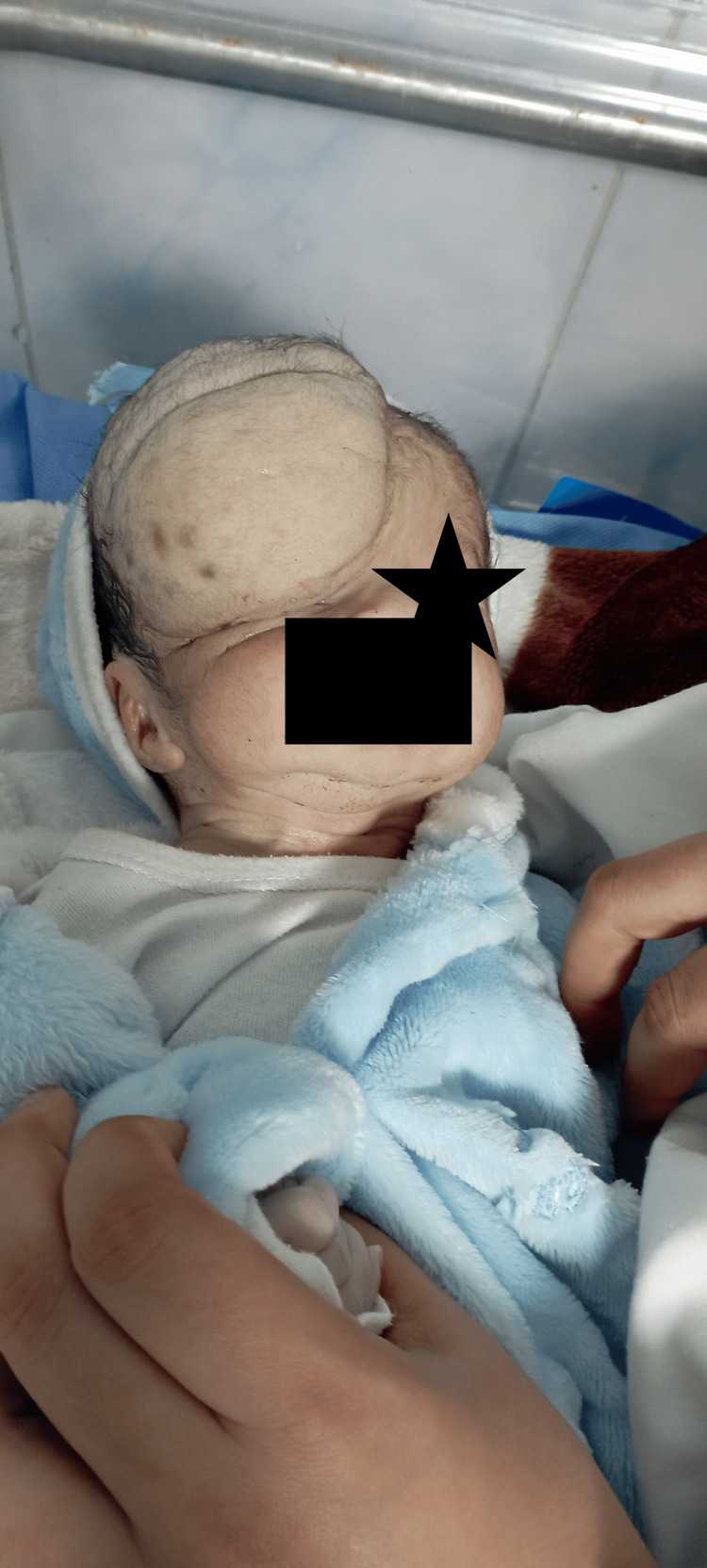
Postnatal clinical appearance of the newborn with cervicofacial lymphangioma.

Continuous fetal heart rate monitoring was reactive and reassuring with appropriate uterine contractions. After five hours of labor, the patient delivered vaginally without an episiotomy or vaginal tears. The postpartum period was uncomplicated (Figure 1).

The newborn was examined by the pediatric team and was found to be pink, reactive, and normotonic with preserved reflexes. Blood glucose was measured at 0.52 g/L. The infant was discharged with the mother and scheduled for pediatric surgical evaluation and excision of the cervical cystic mass at six months of age, along with cardiologic follow-up for congenital heart abnormalities.

Histopathological examination after surgical excision confirmed the diagnosis of cystic lymphangioma.

## Discussion

Cystic lymphangioma is a benign vascular malformation composed of cystic spaces lined by lymphatic endothelium and filled with lymphatic fluid, occasionally mixed with blood. Although it predominantly affects the cervicofacial region in pediatric patients, deep or atypical localizations have also been described.

The pathogenesis remains incompletely understood [5]. The most widely accepted theory suggests a developmental defect resulting from the failure of communication between primitive lymphatic sacs and the venous system. Antenatal diagnosis is mainly based on ultrasonography, which detects approximately two-thirds of cases [3].

On prenatal ultrasound, cystic lymphangiomas usually appear as multiloculated cystic masses with thin septa and variable-sized anechoic cavities. Differential diagnoses include cervical teratoma, hemangioma, branchial cleft cyst, and encephalocele.

Several studies have reported associations between cystic hygroma and chromosomal abnormalities such as Turner syndrome, trisomy 21, trisomy 18, and trisomy 13 [6]. In our case, amniocentesis could not be performed due to financial constraints. The coexistence of CMV infection and multiple cerebral anomalies complicated the prenatal evaluation.

The natural history of cystic lymphangioma is characterized by progressive enlargement due to lymph accumulation. Complications may include hemorrhage, infection, respiratory distress, dysphagia, and cosmetic sequelae.

Management depends on lesion size, localization, symptoms, and associated anomalies. Surgical excision remains the standard treatment for accessible lesions, although complete resection may be difficult because of infiltration into surrounding tissues. Alternative therapies such as sclerotherapy have also shown promising results [7].

The prognosis varies according to lesion extension and associated malformations. Early prenatal diagnosis allows optimized counseling, multidisciplinary planning, and neonatal management.

## Conclusions

Congenital cystic lymphangioma is a rare benign lymphatic malformation that can be diagnosed prenatally using obstetrical ultrasonography. Cervicofacial localization is the most frequent presentation. The association with congenital infections and multiple fetal anomalies requires careful prenatal surveillance and multidisciplinary management. Histopathological examination remains essential for definitive diagnosis. Early recognition and appropriate surgical management can improve neonatal outcomes.
